# Value of quantitative magnetic resonance imaging T1-relaxometry in predicting contrast-enhancement in glioblastoma patients

**DOI:** 10.18632/oncotarget.18612

**Published:** 2017-06-27

**Authors:** Elke Hattingen, Andreas Müller, Alina Jurcoane, Burkhard Mädler, Philip Ditter, Hans Schild, Ulrich Herrlinger, Martin Glas, Sied Kebir

**Affiliations:** ^1^ Neuroradiology, Department of Radiology, University Hospital Bonn, Bonn, Germany; ^2^ Philips GmbH, UB Healthcare, Hamburg, Germany; ^3^ Division of Clinical Neurooncology, Department of Neurology, University of Bonn Medical Center, Bonn, Germany; ^4^ Stem Cell Pathologies Group, Institute of Reconstructive Neurobiology, Bonn, Germany; ^5^ Clinical Cooperation Unit Neurooncology, MediClin Robert Janker Klinik, Bonn, Germany; ^6^ Division of Clinical Neurooncology, Department of Neurology, University of Essen Medical Center, Essen, Germany

**Keywords:** T1-mapping, quantitative MRI, BBB damage, glioblastoma, T1-relaxometry

## Abstract

**SUMMARIZING THE IMPORTANCE OF THE STUDY:**

The repetitive usage of gadolinium-based contrast agents (GBCA) is critical for magnetic resonance imaging (MRI) evaluation of tumor burden in glioblastoma patients. It is also a crucial tool for determination of radiographical response to treatment. GBCA injection, however, comes with a 2.4% rate of adverse events including life-threatening conditions such as nephrogenic systemic fibrosis (NSF). Moreover, GBCA have been shown to be deposited in brain tissue of patients even with an intact blood-brain barrier (BBB). The present study explores quantitative T1 relaxometry as an alternative non-invasive imaging technique detection of tumor burden and determination of radiographical response. This technique exploits specific properties of brain tissue with impaired BBB. With a sensitivity and specificity as high as 86% and 80%, respectively, quantitative T1-relaxometry allows for detecting contrast-enhancing areas without the use of GBCA. This method could make it unnecessary to subject patients to the risk of adverse events associated with the use of GBCA. Nonetheless, a large-scale analysis is needed to confirm our findings.

**Background:**

Gadolinium-based contrast agents (GBCA) are crucial for magnetic resonance imaging (MRI)-based evaluation of tumor burden in glioblastoma (GBM). Serious adverse events of GBCA, even though uncommon, and gadolinium deposition in brain tissue could be avoided by novel imaging techniques not requiring GBCA. Altered tissue composition in areas with impaired blood-brain-barrier also alters the quantified T1 relaxation time (qT1), so that qT1 analysis could replace GBCA-based MRI for the analysis of tumor burden and response.

**Methods:**

As a part of a prospective pilot MRI-relaxometry trial, patients with newly-diagnosed GBM who relapsed under standard radiochemotherapy were selected for this study. At recurrence, subtraction of qT1 maps pre and post-GBCA application (ΔqT1 maps) was used to determine areas of contrast-enhancement. With the contrast-enhancement on ΔqT1 maps as reference, ROC analysis served to detect an optimal qT1 cut-off on qT1 maps prior to GBCA to distinguish between contrast-enhancing tissue and its surroundings.

**Results:**

Ten patients were included. A qT1 value >2051ms predicted contrast-enhancing tumor tissue with a sensitivity of 86% and specificity of 80% (AUC, 0.92; p<0.0001). Interestingly, qT1 prolongation >2051 ms that did not overlap with contrast-enhancing area transformed into contrast-enhancement later on (n=4).

**Conclusion:**

T1-relaxometry may be a useful technique to assess tissue properties equivalent to contrast-enhancement without the need for GBCA application. It may also provide information on sites with future tumor progression. Nonetheless, large-scale studies are needed to confirm these findings.

## INTRODUCTION

Contrast-enhanced magnetic resonance imaging (MRI) uncovers impaired blood-brain barrier (BBB) associated with aggressive features of glioblastoma and is therefore widely used in the diagnosis and monitoring of this highly malignant brain tumor. A significant increase of the contrast-enhancing area is the most relevant criterion for diagnosing tumor progression and may indicate a need to switch therapy [1–3]. MRI contrast agents used for monitoring tumor burden of glioblastoma patients are based on gadolinium (GBCA). Apart from the discomfort for the patient prompted by the intravenous injection itself, prolonged scanning time, and additional costs, severe adverse events have been observed with GBCA usage. Adverse events upon GBCA application are uncommon with a frequency of 2.4% [4]. However, some of them, such as the nephrogenic systemic fibrosis (NSF) might impede GBCA usage especially in patients with renal failure, which are particularly prone to gadolinium-induced NSF [4]. Further, recent studies reported gadolinium deposition in the brain in patients receiving repetitive GBCA injections [5], even in individuals with intact BBB. Last but not least, gadolinium has a negative environmental impact, it is a costly rare earth metal with limited natural resources while its use and consequent excretion contaminates water [5–7]. Limiting GBCA usage would therefore be advisable but GBCA are particularly important in patients with diseases like glioblastoma who need repetitive GBCA-based MR examinations. For these patients, GBCA-independent new imaging techniques need be developed.

In the brain, the water content is strictly regulated by the BBB that preserves the central nervous system homeostasis. In MRI, the T1-relaxation time is a basic physical value which is substantially influenced by the content of interstitial tissue water [8–10]. When the tumoral BBB is damaged, water and paramagnetic gadolinium (Gd) complexes may pass into the perivascular tissue, allowing thermodynamic interaction with their surroundings, the lattice. The spin-lattice relaxation time, also known as T1, decreases when proteins and Gd complexes accumulate but increases when only water accumulates [11–13]. While Gd complexes are too large to diffuse over long distances outside the tumor tissue, water diffuses far beyond the tumor tissue and results in peritumoral edema mainly along white matter tracts [14]. Increased tissue water results in T1-prolongation in glioblastoma tissue and its surrounding edema [12, 13]. However, different composition of the lattice according to the presence of tissue water should result in different T1 values. QT1 mapping is a quantitative method that enables estimation of the interstitial water content [15, 16].

We suggest that there is a threshold of T1-increase in pre-contrast images that indicates the presence of enhancing tumor tissue and discriminates it from non-enhancing peritumoral area and from normal brain tissue. To evaluate this hypothesis, we measured T1 in patients with histologically proven glioblastoma (GBM) pre- and post-injection of GBCA. Areas with significant T1 shortening post-GBCA served to detect contrast-enhancing tumor tissue. Comparing pre-GBCA T1 in the so obtained contrast-enhancing tumor tissue against pre-GBCA T1 in the surrounding tissue served to determine a potential cut-off value for discriminating tumor tissue from peritumoral edema and normal brain tissue.

## RESULTS

### Subjects

Ten patients were eligible for inclusion in this pilot study. The corresponding patients’ characteristics are provided in Table 1.

**Table 1:** Patients’ and tumor characteristics

Pt. no.	Sex	Age^1^	First-Line Treatment	MGMT	TP	Extent of overlap between area with qT1 >2051 ms and enhancing tumor	Extent of non-overlapping area with qT1 > 2051 ms later transforming to enhancing tumor^5^	qT1 (ms)	TP of progression (RANO)
1	f	53	RT*+TMZ, TTF, D	-	TP9	excellent	completely	2481	
					TP10	excellent	completely	2482	
					TP11	motion artifacts		2250	TP11
2	f	58	B, RT+TMZ	+	TP1	excellent	none	2158	
					TP2	excellent	completely	2676	
					TP3	excellent		2605	TP3
3	f	62	pR, RT+TMZ	-	TP4	excellent	none	2772	
					TP5	moderate	none	2598	
					TP6	moderate		2083	TP 6; necrotic tumor
4	m	67	pR, RT+TMZ	-	TP2	moderate	partially (80%)	2615	
					TP3	excellent	partially (60%)	3051	
					TP4	excellent		3091	TP4
5	m	74	pR, RT+TMZ, D	+	TP0	excellent	completely	2076	
					TP1	excellent	completely	2227	
					TP2	excellent		3631	TP2
6	m	58	B, TR+TMZ	-	TP2	excellent	partially (90%)	2804	
					TP4	excellent	none	2776	
					TP5	excellent		2955	TP5
7^2^	m	60	cR, RT+TMZ	-	TP3	excellent	none	2911	
					TP4	excellent	none	3088	
					TP5	excellent		2743	TP6
8	f	72	cR, RT, TP3: TMZ	+	TP3	excellent	none	1994	
					TP4	excellent	none	2705	
					TP5	moderate^4^		1962	TP5
9	f	69	pR, RT+TMZ	-	TP2	moderate	none	2516	
					TP3	moderate	none	2928	decreased enhancement
					TP4	poor		1842	TP4; necrotic tumor
10^3^	n	47	cR, RT+TMZ, adjuvant CCNU+TMZ	+	TP7	poor	none	1873	
					TP8	poor	none	1660	
					TP9	poor		1517	TP9; re-surgery: pseudo-progression

^1^, Age at first glioblastoma surgery; ^2^, gliosarcoma; ^3^, glioblastoma with oligodendroglial component; ^4^, T1 non-prolonged area regressed at TP6; ^5^, extent of area with qT1 > 2051ms that does not overlap with contrast-enhancing area, which transforms to enhancing tumor on a later follow-up

f, female; m, male; MGMT, O6-Methyl-Guanin-Methyl-DNA-Transferase promoter methylation; +, methylated; -, not methylated; TP, time point; B, stereotactic biopsy; cR, complete resection; pR, partial resection; RT, radiation therapy with 60 Gy; RT*, radiation therapy with 22 Gy; TTF, treatment with tumor treating fields; TMZ, temozolomide; RT+TMZ, combined radiochemotherapy with TMZ followed by adjuvant TMZ; CCNU, chloroethyl-cyclohexyl-nitrosourea; D, dexamethasone treatment as of surgery

### Defining a cut-off value for pre-GBCA qT1

**Figure 1:**
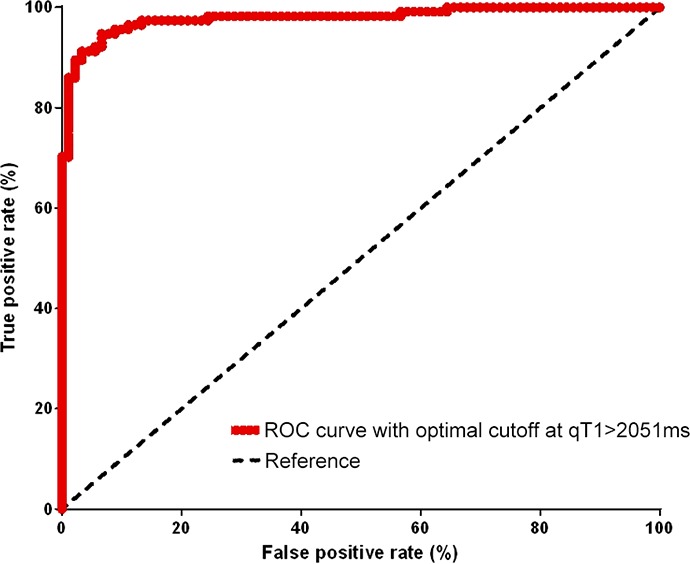
ROC curve This plot shows qT1 values [ms] at different cutoffs and quantifies their the sensitivity and specificity in discriminating the solid contrast-enhancing tumor from the surroundings. The optimal performance is reached at a qT1 value of 2051ms.

ROC analysis (Figure 1) revealed that a pre-GBCA qT1 value of >2051 ms discriminated best between the contrast-enhancing tumor on ΔqT1 maps and the surrounding tissue. Pre-GBCA qT1 values above 2051 ms predicted true tumor tissue with a sensitivity of 86% (95% confidence interval (CI), 78 – 92%) and a specificity of 80% (CI, 71 - 87%). The area under the ROC curve (Figure 1) was 92% ± 1.8% standard deviation (SD) (CI, 89 - 95%; p<0.0001).

### Overlap between areas with elevated pre-GBCA qT1 and contrast-enhancing tumor

The overlap between areas with pre-GBCA qT1 >2051 ms (qT1-prolonged area) and the contrast-enhancing tumor on ΔqT1 maps (gold standard) was on average 85% across all time-points and patients (Table 1). We observed a varying extent of overlap, which was subdivided in three major extent of overlap classes: excellent (≥ 90%), moderate (50-89%) and poor overlap (0-49%).

**Figure 2:**
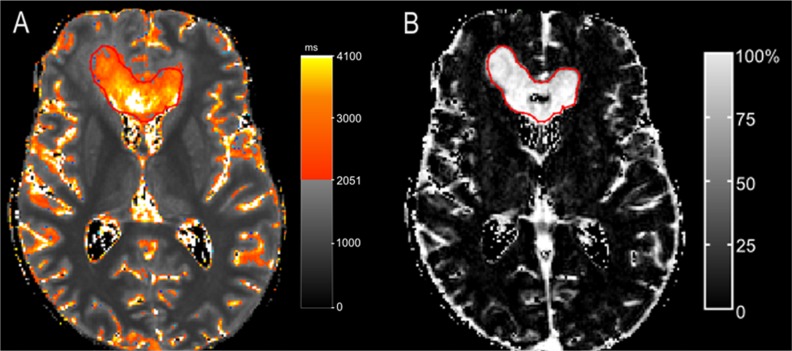
A patient with >90% overlap (patient 2) The color map **(A)** shows areas with pre-GBCA qT1 > 2051 ms, delineated by a red line as a gradient between red and yellow. A nearly complete overlap is seen at time-point of progression between pre-GBCA qT1 (A) and the contrast-enhancing tumor on the subtraction map ΔT1, which is outlined in red (pre-GBCA qT1 – post-GBCA qT1) **(B)**.

An excellent overlap was observed at least one of three evaluated time-points in eight patients. Moreover, seven patients showed even an excellent overlap at two or more time-points (Table 1, patients 1,2,4,5,6,7,8). As an example, Figure 2 shows the qT1 maps of one of those patients (Table 1, patient 2).

**Figure 3:**
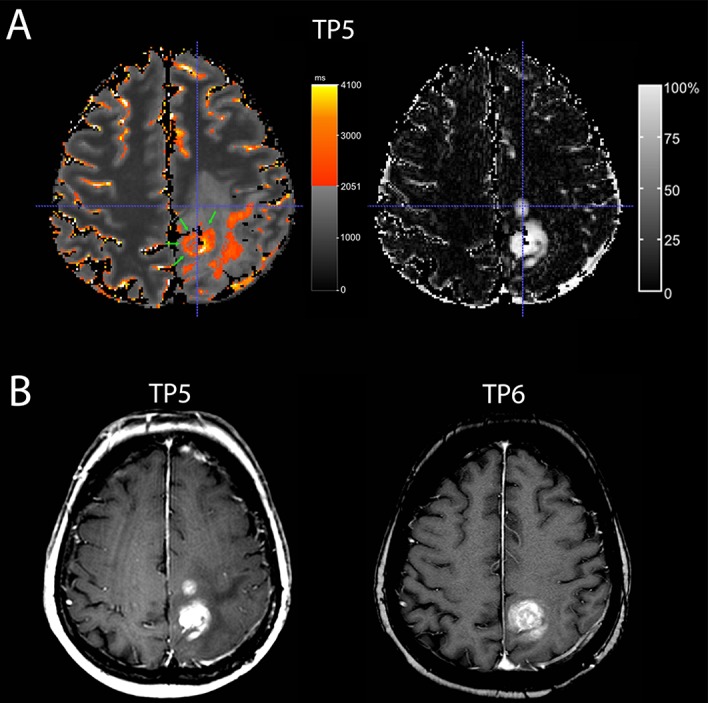
A patient with moderate overlap (patient 9) A Quantitative map with a subtraction map qT1 at time-point TP 5) **(A)** and conventional MRI at TP 5 and TP 6 **(B)** of a 70-year-old woman with recurrent GBM. At TP 5, the quantitative map (A, on the left-hand side) shows pre-GBCA qT1 area with >2051 ms (red to yellow, green arrows) with a good overlap of the dorsal contrast-enhancing tumor seen on the subtraction map ΔT1 (A, on the right-hand side), but the second smaller contrast-enhancing tumor area (crosshair) is missing on the color map. This contrast-enhancing area without overlap (crosshair) regressed in the next conventional MRI (TP 6) (B), whereas the contrast-enhancing tumor with overlap progressed. Also, note additional area of T1-prolongation outside the contrast-enhancing tumor.

A moderate overlap was found at least one time-point in four patients (Table 1, patients 3,4,8,9). A predominantly moderate overlap, i.e. a moderate overlap at least 2 of 3 time-points, however, was only seen in two patients (Table 1, patients 3,9). As evidenced by the respective ΔqT1 maps (gold standard), those two patients had mainly necrotic tumors at the time of progression. In one patient (Table 1, patient 9), the contrast-enhancing area, which did not overlap with the qT1-prolonged area, regressed at the next MRI timepoint, whereas the overlapping contrast-enhancing tumor progressed (Figure 3).

A poor overlap could be demonstrated at least one time-point in two patients (Table 1, patients 9 and 10). In one of those patients (Table 1, patient 10), the contrast-enhancing area had 0% to maximally 10% overlap with the qT1-prolonged area. This patient had progressive disease according to RANO and underwent re-surgery that revealed mainly therapy-induced tissue changes and only very scattered tumor cells so that these changes were classified as pseudoprogression.

### Predictive role of qT1-prolonged areas for tumor recurrence

**Figure 4:**
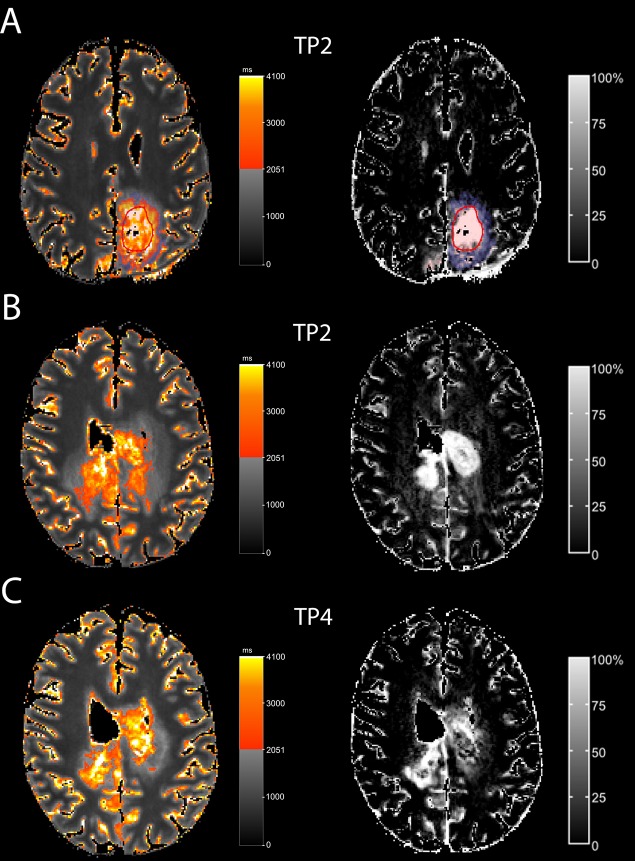
T1-prolongation > 2051 ms outside the solid contrast-enhancing tumor In one patient (A; patient 5), T1-prolongation >2051 ms outside the solid contrast-enhancing tumor (red circle) partially fitted with the subtle enhancement (ΔqT1, blue). In another patient (B, C; pat 6), T1-prolongation >2051 ms outside the solid contrast-enhancing tumor at TP 2 (B) partially matched subtle enhancement (ΔqT1). At TP4 (C), this area transformed to contrast-enhancing tumor.

In five patients (Table 1, patients 2,3,7,8,9), the qT1-prolonged area was restricted to the contrast-enhancing tumor. In the remaining five patients, the qT1-prolonged area extended beyond the contrast-enhancing tumor (Figure 3 and 4 show this in exemplary fashion for patients 5 and 6). The qT1-prolonged area outside the contrast-enhancing tumor partially matched the subtle contrast-enhancement surrounding the solid contrast-enhancement (Figure 4A shows this in exemplary fashion for patient 5). Small parts of these areas transformed to contrast-enhancing tumor at the time of progression in four patients (Patients 1,4,5,6) (Figure 4B and 4C show this in exemplary fashion for patient 6), or at a later timepoint (n=1, patient 4). Interestingly, three of the five patients (Patients 1,4,5) with the qT1-prolonged area outside the contrast-enhancing tumor had prior partial resection or biopsy with substantial remaining tumor burden.

## DISCUSSION

This work shows that the quantitative relaxation time T1, qT1, is markedly increased in contrast-enhancing areas of glioblastoma. On pre-GBCA maps, areas with qT1 >2051 ms predicted the contrast-enhancing tumor with a good diagnostic performance. Thus, without the need for GBCA application, the tumor may probably be made visible on pre-GBCA maps by identifying areas with a qT1 increase above 2051 ms. Consequently, qT1 maps might be a useful tool to visualize and monitor tumor growth without using a contrast agent. This might be particularly important in preventing contrast agent induced adverse events.

In the present study, we observed high values of qT1 in the contrast-enhancing tumor. T1-relaxation time is known to increase when water accumulates in the absence of Gd complexes [11–13]. There may be two major reasons for this finding. First, tumor tissue consists of tumor cells, activated glia cells, tumor vessels, and micro-necrosis, which all interact to a very small degree with the spins of water molecules and thus result in high T1 values [17, 18]. Second, the immediate vicinity of contrast-enhancing tumor to leaky tumor vessels leads to a high water content and thereby high T1 [19]. Our findings are in line with previous reports of increased T1 in brain tumors [20–22].

In contrast, T1 prolongation in peritumoral edema is less pronounced because peritumoral edema mainly extends along the white matter tracts, which are known for a high protein and fat content [23]. These properties allow for qT1 to be used as a discriminator between true tumor tissue and peritumoral edema.

Studies carried out in the 1990s found more variable T1 values in glioblastoma tissue as compared to our data [24, 25]. Beside technical and methodical advances, our results differ from those of previous studies in that we only analyzed T1 in the contrast-enhancing part of the tumor, which was semi-automatically segmented on ΔqT1 maps. ΔqT1 maps that were calculated on the grounds of quantitative T1 values pre- and post-GBCA, allowed us to exclude macroscopically visible necrotic areas, which contain various elements such as hemorrhages and protein-rich exudates that shorten T1 values.

Another major finding of this study is that areas with qT1 values lower than 2051 ms may represent pseudoprogression instead of true tumor progression. Although we aimed to include only patients with unequivocal tumor progression according to RANO, there was one patient with late tumor-imitating therapy-induced changes, confirmed via biopsy, and one patient with partial regression of the contrast-enhancing areas. In both patients, qT1 values were below 2051 ms. This shows that classification by RANO may not always be suited to differentiate between pseudoprogression and true tumor progression. QT1 measurements may be superior in this regard.

Color-coded visualization of areas with qT1 increase >2051 ms did not show satisfactory results in all patients. In patients with predominantly necrotic tumor, the necrotic area hardly overlapped with the contrast-enhancing tumor. In these patients, areas of contrast-enhancing tumor were missed on pre-GBCA qT1 maps based on a qT1 cut-off value of 2051ms. In addition, patients with substantial tumor burden also exhibited qT1-prolongation outside the contrast-enhancing tumor. The biological value of these areas remains questionable because only smaller parts of these areas transformed into tumor later on. A putative explanation for the low qT1 values observed in the necrotic area might be the elevated protein and lipid content present in necrotic tissue [26], which is usually accompanied by decreased qT1. A comparison of areas with decreased qT1 with the respective histological phenotype should be included in future studies to help explain our observations.

A major limitation of this pilot study is the small sample size. Thus, statistical interpretation is restricted and our findings need to be corroborated in an independent trial with a larger sample size. In addition, translation into clinical application has several limitations: First, the longer measurement times of quantitative MRI and, to avoid missing the critical time-point of tumor progression, short time intervals of 6 weeks were only tolerable for a limited number of patients. Second, this pilot study included patients after surgery and during therapy. Therefore, the contrast-enhancing areas observed on ΔqT1 maps may reflect therapy-induced changes to some extent. To account for this issue, we only included patients with progressive disease and included the evaluation of qT1 maps before the time-point of RANO-defined tumor progression. In the clinical setting of a tertiary referral hospital, however, many patients are admitted with external MR images. Obtaining pre-surgery qT1 maps would mean an additional MR session, which is time-consuming and may cause discomfort for the patients. Therefore, this pilot study is primarily intended to focus on the feasibility and diagnostic value of qT1 mapping in patients with pathohistologically proven glioblastoma. Nonetheless, the results are promising and encourage to conduct future studies evaluating this method, particularly in therapy-naïve glioblastoma patients.

Overall, this pilot study uncovers a putative role for qT1 mapping in detecting tumor tissue without using GBCA, a method that could have major implications for daily clinical practice and would help avoid subjecting patients to adverse events evoked by GBCA administration. This study encourages validation in a larger trial.

## MATERIALS AND METHODS

### Subjects

This study is a part of a prospective, non-interventional pilot MRI study of adult patients with histopathologically confirmed primary glioblastoma or gliosarcoma. All patients signed an institutional review board-approved informed consent form prior to enrolment. We monitored these glioblastoma patients with MRI at 6-week intervals after tumor surgery and during standard therapy (according to the Stupp protocol, [3]). From the whole cohort, we selected those GBM patients with at least five monitoring MRIs and progressive disease according to the Response Assessment in Neuro-Oncology Criteria (RANO, [2]) to ensure that only patients with true progression and not pseudoprogression were analyzed.

### MRI study protocol

MRI studies were performed at a 3.0 Tesla whole body system MRI (Achieva, Philips Healthcare, The Netherlands) with an 8-channel phased array head coil. Standard MRI included Fluid-attenuated inversion recovery pulse sequences (FLAIR) and T1-weighted (T1w) spin echo sequences pre- and post-injection of GBCA.

The qT1 data were derived by means of an isotropic 3D ultrafast gradient echo (TFE; 1×1×1 mm^3^ resolution, field of view of 240 × 220 mm^2^, 120 slices) using radial turbo direction with a profile order of low-to-high for more efficient readout speed and an adiabatic inversion preparation (hyperbolic secant pulse) with five consecutively varying inversion delays (150, 350, 750, 1200, 2300 ms) and parallel imaging. The shot interval (SI) was fixed to 3000 ms with an efficient TFE shot-length of 660 ms (TFE-factor = 105) for the five various inversion delays. Accuracy in the desired clinical T1-range was verified with a phantom containing 12 flasks of distilled water doped with NiCl2.

T1-relaxation times (qT1) were calculated before and following administration of a GBCA (Gd-DO3A-butrol of 0.1 mmol/kg body weight).

### Data analysis

In a first step, we generated qT1 maps and subtracted qT1 maps pre-injection of GBCA (pre-GBCA qT1) from respective post-GBCA maps. On these subtraction maps (ΔqT1maps), the contrast- enhancing part of the tumor became visible as a result of T1 shortening. On the ΔqT1 maps, we observed a pronounced, solid contrast-enhancing region corresponding to the enhancement visible on T1w images and a surrounding subtle, cloudy enhancement not visible on conventional T1w images, the latter of which might represent diffuse angiogenic tumor invasion [27]. To differentiate this subtle enhancement from “solid” contrast-enhancing tumor, we introduced a threshold of >50% T1-shortening post-GBCA, which matched well the contrast-enhancing tumor on conventional post-GBCA T1w images.

In a second step, we searched for a reliable pre-GBCA qT1value that may discriminate the solid contrast-enhancing tumor from its surroundings. We used this value as a lower threshold for a color scale, with which we visualized the tumor. We then compared the threshold-based tumor area on pre-GBCA qT1 maps against the “gold standard” contrast-enhancing tumor on ΔqT1 maps.

### qT1 maps

We calculated T1-relaxation time maps from the IR-magnitude data with a fixed likelihood estimate for the goodness of the inversion pulse (F=2.0), also accounting for incomplete longitudinal relaxation at the next excitation. Maps were generated by an in-house script in Matlab (release 2014a, MathWorks Inc). We then scaled these maps to obtain similar T1 values in the normal appearing white matter both before and after contrast agent application, which improved the visibility of regions with decreased T1 values after injection of GBCA. Finally, qT1 maps from all time-points of a given patient were then linearly coregistered with reg_aladin (Translational Imaging Grup, http://cmictig.cs.ucl.ac.uk/wiki/index.php/Reg_aladin) to the very first pre-GBCA qT1 map of that patient.

### qT1 subtraction maps for contrast-enhancing tumor

Out of the coregistered qT1 maps pre-injection and post-injection of GBCA, we generated subtraction ΔqT1 maps as follows:

$$\text{subtraction map}(\text{qT}1)=100*\frac{\left|T1(\text{pre}-\text{GBCA})-T1(\text{post}-\text{GBCA})\right|}{T1(\text{pre}-\text{GBCA})}$$

On ΔqT1 maps, we defined the contrast-enhancing tumor as regions with >50% T1 shortening after GBCA. To this end, we used the “thresholding” drawing mode of the ITK-SNAP software, which expands manually set seed regions based on an active contour algorithm [28]. For each subject, we placed a small seed within the visible contrast-enhancement and allowed the algorithm to expand this initial contour to the surrounding regions (with >50% T1 shortening) until the final contour encompassed all the visible contrast-enhancement. In cases where the automatically defined contour extended to and beyond the vessels around the tumor, we manually excluded those vessels from the final region of interest.

### Overlap between tumor on pre-GBCA qT1 maps and contrast-enhancing tumor

To detect the tumor on pre-GBCA qT1, we encoded any prolongation of qT1 above the predefined cut-off value as a color scale from red to yellow in the ITK-Snap program (http://www.itksnap.org). This assignment allowed rapid visualization of regions with significantly prolonged qT1 and comparing it against the contrast-enhancing tumor on ΔqT1 maps.

Drawing and active contour segmentation tools in ITK-Snap make it possible to easily delineate the area of contrast-enhancing tumor on ΔqT1 maps (gold standard). Using the program's crosshair tool in the drawing mode, the area of contrast-enhancing tumor was compared to the area of qT1 prolongation above the cut-off value on pre-GBCA maps. Then, the percentage overlap – in steps of 10% - was documented and the extent of overlap was categorized into

1. excellent overlap: defined as ≥ 90% overlap
2. moderate overlap: defined as 50-89% overlap
3. poor overlap: defined as 0-49% overlap

To ensure that only true tumor cases were included and to avoid the inclusion of cases of pseudoprogression, we restricted the analysis of time-points to those where at least two previous time-points showed no pseudoprogression.

We further analyzed the residual pre-GBCA qT1 prolongation that did not overlap with the contrast-enhancing tumor. This was done to evaluate whether the non-overlapping areas convert to a contrast-enhancing tumor in a later follow-up MRI. To this end, pre-GBCA qT1 and ΔqT1 maps were compared with those at later time-points.

### Statistics

In each patient and at each time-point, we calculated the pre-GBCA qT1 values within the contrast-enhancing tumor and within its surroundings. Receiver operating characteristics (ROC) analysis served to determine the optimal cut-off value of pre-GBCA qT1 that discriminates best between pre-GBCA qT1 values of the contrast-enhancing tumor and pre-GBCA qT1 values of its surrounding tissue.
